# Iatrogenic Nerve Injuries in Head and Neck Surgeries: A Systematic Review of Mechanisms, Outcomes, and Prevention Strategies

**DOI:** 10.7759/cureus.101768

**Published:** 2026-01-18

**Authors:** Aymen J Mohamed, Ruba Mohamedahmed, Mariem Aboussaid, Rayan Adam Mahdi Edrees, M-Malek A Alghawee, Asim Ahmed

**Affiliations:** 1 General Practice, Saudi German Hospital, Medina, SAU; 2 Surgery, Al Borgaig Hospital, Khartoum, SDN; 3 Surgery, Cadi Ayyad University, Faculty of Medicine and Pharmacy of Marrakech, Marrakech, MAR; 4 Surgery, Jazan General Hospital, Jazan, SAU; 5 General Practice, Umm Al Quwain Hospital, Sharjah, ARE; 6 Epidemiology, University of Gezira, Khartoum, SDN

**Keywords:** facial nerve, head and neck surgery, iatrogenic nerve injury, recurrent laryngeal nerve, surgical complications

## Abstract

Iatrogenic nerve injury in head and neck surgery remains a substantial yet potentially preventable source of morbidity across endocrine, otolaryngologic, and related procedures. This systematic review synthesized evidence to identify the nerves most frequently affected, delineate operative mechanisms of injury, describe the clinical course, and evaluate preventive strategies. Traction on visually intact nerves emerged as the predominant mechanism, with additional contributions from thermal injury, compression, ischemia, and entrapment by suture or clip. The recurrent laryngeal, facial, trigeminal (inferior alveolar and lingual), spinal accessory, and lower cranial nerves were identified as the principal structures at risk, with procedure type influencing the pattern and severity of deficits. Prevention centered on deliberate visual identification, meticulous dissection along natural planes, and risk-stratified use of intraoperative nerve monitoring, which proved most beneficial in complex or reparative fields. While most postoperative deficits resolved over time, a subset persisted, impairing voice, swallowing, facial movement, shoulder function, or orofacial sensation. Early, tension-free microsurgical repair was associated with superior recovery compared to delayed intervention. Despite heterogeneity in definitions, assessment timing, and follow-up, these findings support a practical prevention framework that integrates precise anatomical techniques, gentle handling, structured monitoring in high-risk cases, and timely referral for persistent deficits to minimize avoidable nerve injury and improve long-term outcomes.

## Introduction and background

Iatrogenic nerve injuries in head and neck surgery, while sometimes unavoidable, can cause substantial morbidity ranging from transient dysfunction to permanent deficits such as facial paralysis, dysphonia, and chronic neuropathic pain [1]. Risk increases with the dense anatomy of the region, where cranial nerves course in close proximity to common surgical planes, which increases the likelihood of traction or transection injury [2]. The facial nerve is particularly vulnerable in parotid surgery, with reported rates of permanent facial palsy ranging from approximately 2% to 6%, and immediate postoperative weakness between 14% to 65% [3,4]. The recurrent laryngeal nerve (RLN) is at risk during thyroidectomy, with transient injury rates of 2% to 11% and permanent injury rates of 0.6% to 1.6%, and remains a leading cause of postoperative voice disorders when injured [5]. During neck dissections, the spinal accessory and marginal mandibular nerves are frequently affected; reported spinal accessory nerve (SAN) injury can be very high in radical dissections, reaching up to 94.8%, while marginal mandibular nerve injury is estimated at around 12.7% to 13.1% in modified radical and selective dissections [6]. Mechanisms span traction or stretch, compression, devascularization, and thermal damage, each producing distinct pathophysiology and clinical sequelae [7]. Heterogeneity in mechanisms, techniques, and patient comorbidities drives variability in reported incidence and complicates standardization of prevention.

**Figure 1:**
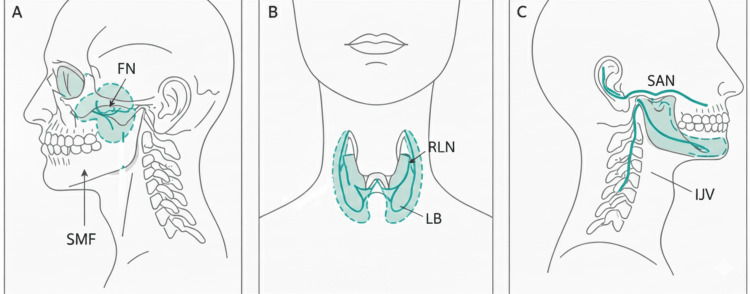
Nerves at risk in head and neck surgery. Anatomical illustration of nerves at risk. A: Parotidectomy. B: Thyroidectomy. C: Neck dissection. FN: facial nerve; SMF: stylomastoid foramen; RLN: recurrent laryngeal nerve; LB: ligament of Berry; SAN: spinal accessory nerve; IJV: internal jugular vein. Image created by the author.

The most commonly injured nerves and their index procedures, emphasizing the anatomical danger zones across parotid, thyroid, and neck dissections, are illustrated in Figure 1.

This review synthesizes evidence on the prevalence, mechanisms, and outcomes of iatrogenic nerve injuries across head and neck procedures, evaluating preventive strategies such as intraoperative nerve monitoring, meticulous exposure, and anatomical technique to inform surgical safety and patient counseling [8]. By integrating data across multiple specialties, the review also summarizes the most frequently injured nerves and their corresponding procedures, collating both short- and long-term outcomes to inform postoperative expectations and rehabilitation.

A structured synthesis was particularly warranted to address fragmented data and variable methodologies that characterize single-center or nerve-specific studies. Prior literature highlights that sensory dysfunction after orthognathic surgery is often attributed to direct or indirect trigeminal injury [9]. At the same time, neck dissection series consistently implicate the spinal accessory and marginal mandibular nerves, with the risk influenced by the extent and technique of the dissection [6]. Across mandibular procedures, the lingual and inferior alveolar nerves emerge as high-risk structures, underscoring the need for focused preventive measures and targeted surgical training. Additionally, this review aggregates patient- and procedure-level risk factors to support preoperative risk stratification and informed counseling. Management of late paresthesia, particularly in tissues innervated by the mandibular division, encompasses a range of conservative pharmacologic strategies to microsurgical repair, with selection guided by etiology, timing, and deficit profile [10].

Preoperative assessment helps identify high-risk patients and supports individualized planning, including awareness of specific scenarios such as lingual nerve (LN) injury related to airway management. Intraoperatively, attention to anatomic variation, deliberate visual identification of at-risk nerves, and surgical experience remain key to minimizing iatrogenic injury [8]. In oral and maxillofacial surgery, the inferior alveolar nerve (IAN) is particularly vulnerable during third molar extraction and dental implant placement, with reported injury rates of 0.4% to 8%, while permanent injury is typically under 1% [11]. Lingual nerve injury rates during similar procedures are of comparable magnitude in many reports. Overall, the mandibular division of the trigeminal nerve is more susceptible to injury than the ophthalmic or maxillary divisions. The IAN is most frequently injured, followed by the lingual nerve, particularly during third molar surgery, sagittal split ramus osteotomy, endodontic therapy, and implant placement [12]. Temporary lingual somatosensory disturbance is reported in roughly 0.6% to 2.0% of mandibular third molar extractions.

In contrast, permanent injury is rare, with most cases linked to third-molar surgery, periodontal procedures, and mandibular implants [13]. IAN injuries can produce dysesthesia in about 0.4-8% of cases, with permanent deficits uncommon, yet even transient deficits may meaningfully affect speech, mastication, and psychosocial well-being. In addition to surgery, local anesthetic injections can cause trigeminal nerve injury, likely via needle trauma or anesthetic neurotoxicity [14]. Other potential etiologic factors include pre-prosthetic surgery, various orthognathic surgical procedures, ablative tumor surgery involving mandibular resections, osteoradionecrosis, osteomyelitis, and maxillofacial trauma [12].

## Review

Materials and methods

This review adhered to the Preferred Reporting Items for Systematic Reviews and Meta-Analyses (PRISMA) 2020 guidelines [15] and was prospectively registered on the International Prospective Register of Systematic Reviews (PROSPERO). A protocol finalized a priori specified the objectives, eligibility criteria, outcomes, and synthesis methods, and was followed without deviation except where noted under amendments. The review question examined across head and neck procedures the prevalence and mechanisms of iatrogenic nerve injury, the resulting short- and long-term outcomes, and the effectiveness of preventive strategies, particularly intraoperative nerve monitoring.

Eligibility was defined using the PICOS (population, intervention, comparison, outcomes, and study design) framework: human surgical populations in the specified specialties; exposures comprising standard operative care, prevention strategies, and relevant anesthesia factors; comparators including usual care or alternative techniques or none for descriptive cohorts; and outcomes led by incidence of nerve injury, with secondary outcomes including mechanisms, functional recovery and time to recovery, voice and swallowing metrics, neurosensory measures, shoulder function, complications, quality of life, neuropathic pain, and prevention effectiveness [16]. Randomized and non-randomized comparative studies, prospective and retrospective cohorts, and case-control designs were included. Case series with fewer than 10 patients were excluded from quantitative synthesis but were considered qualitatively when they uniquely informed mechanisms. Studies of traumatic etiology, purely diagnostic investigations without postoperative nerve outcomes, pediatric-only cohorts not pertinent to operative technique, and animal or cadaveric work were excluded from analysis, with the latter used only for anatomical context when relevant.

Information sources included MEDLINE, Embase, Cochrane CENTRAL, Web of Science, and Scopus from inception to the final search date, supplemented by trial registries and backward citation screening of included articles and relevant reviews to mitigate publication bias. Grey literature was assessed for eligibility, but quantitative synthesis prioritized peer-reviewed full texts to ensure definitional and methodological consistency. A librarian-vetted search strategy combined controlled vocabulary and keywords for iatrogenic injury, target procedures, nerve structures, and prevention terms. No language or date limits were applied.

Study selection proceeded in two stages, with independent reviewers screening titles and abstracts, followed by a full-text review against the PICOS criteria. Disagreements were resolved by consensus or, when necessary, by a third reviewer. Reasons for full-text exclusions were recorded in accordance with the PRISMA guidelines. Data extraction used a piloted form completed independently by two reviewers and reconciled by cross-check, capturing bibliographic details, specialty and country, study design, sample size, nerves at risk, procedure type and extent, intraoperative nerve monitoring use and modality, definitions and ascertainment of transient versus permanent injury, mechanism categories, functional and patient-reported outcomes, time to recovery, adverse events, and funding sources. Study authors were contacted when critical data were missing or inconsistent.

The risk of bias was assessed at the outcome level using the Cochrane Risk of Bias 2 (RoB 2) tool for randomized trials and the Newcastle-Ottawa Scale (NOS) for non-randomized designs, with prespecified thresholds for low, moderate, and high risk. Systematic reviews used for contextualization were assessed using the Assessment of Multiple Systematic Reviews 2 (AMSTAR-2). Effect measures were presented as odds ratios or risk ratios with 95% confidence intervals for dichotomous outcomes, and as mean or standardized mean differences for continuous outcomes. Given anticipated heterogeneity in procedures, ascertainment, and follow-up, a structured narrative synthesis was performed by specialty and nerve, complemented by random-effects meta-analysis when comparisons were methodologically comparable. Heterogeneity was quantified using I², τ², and the Q test. Subgroup analyses and meta-regression were planned for datasets with at least 10 studies. Sensitivity analyses excluded high-risk-of-bias studies or those lacking standardized postoperative nerve assessment and explored estimator effects. Small-study bias was examined with funnel plots and Egger's test, where feasible, and trim-and-fill analysis was considered exploratory. The certainty of evidence for pooled and narrative outcomes was graded using the GRADE (Grading of Recommendations, Assessment, Development, and Evaluation) approach.

Outcome definitions were standardized a priori, defining transient injury as resolution within six months and permanent injury as persisting beyond six to 12 months. Ascertainment quality was flagged when routine laryngoscopy or standardized neurosensory testing was performed, and mechanisms were coded into predefined categories, marking "mixed/unspecified" when applicable. Protocol amendments, such as explicitly including select cervicobrachial procedures that report clinically relevant outcomes related to cranial or cervical nerves due to neck exposure, were finalized before analysis and documented with a rationale.

Study selection

**Figure 2:**
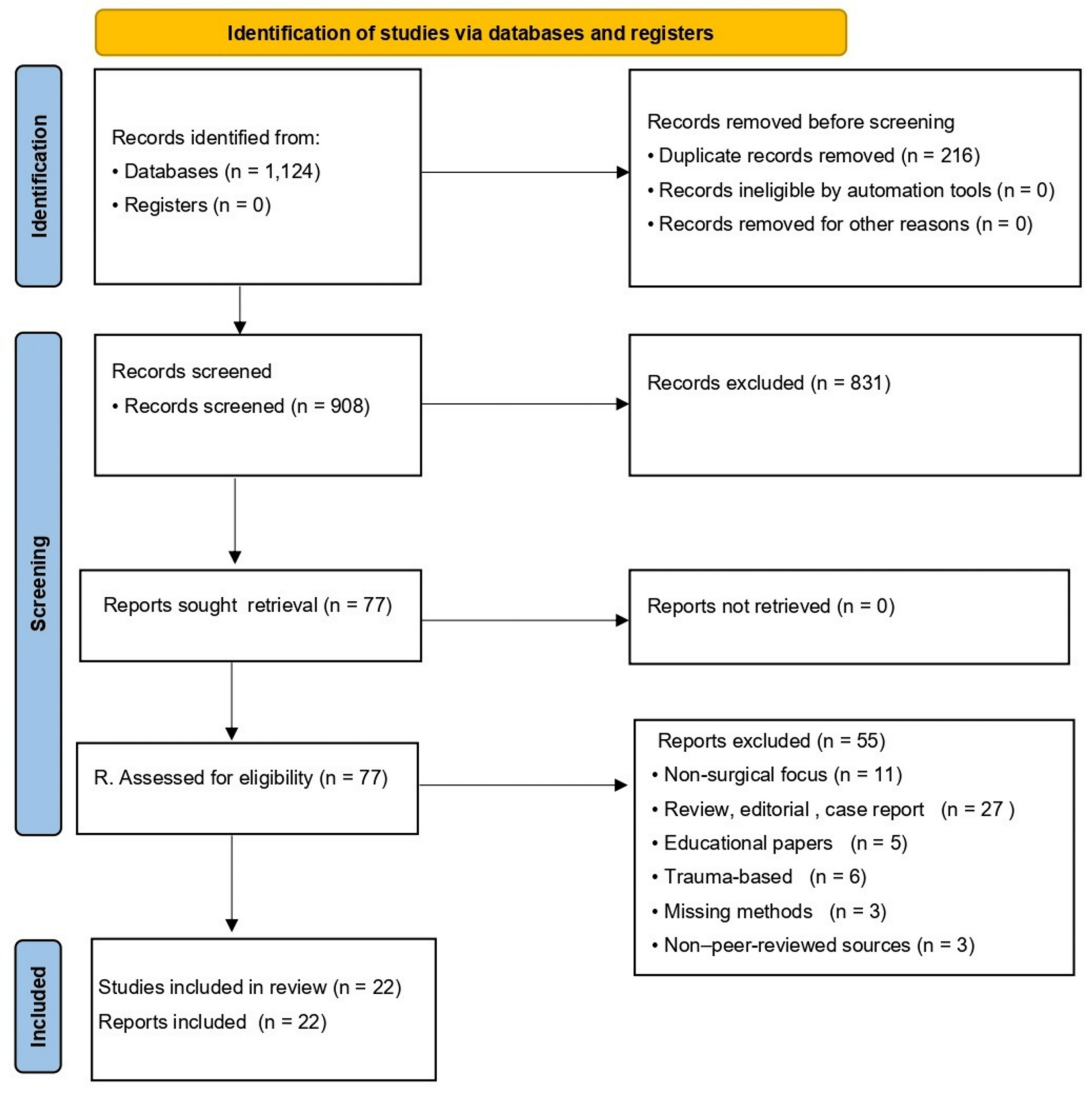
PRISMA (2020) flow diagram of study selection. Flow diagram summarizing the identification, screening, eligibility assessment, and inclusion of studies in this systematic review, based on the PRISMA (2020) reporting guidelines by Page et al. [15]. PRISMA: Preferred Reporting Items for Systematic Reviews and Meta-Analyses; n: number of records.

The PRISMA identified 1,124 records from databases, with no additional records from registers. After removal of 216 duplicates, 908 unique records were screened by title and abstract, and 815 were excluded for irrelevance to PICOS. Ninety-three full-text reports were sought and successfully retrieved for eligibility assessment. Seventy-one reports were excluded for the following reasons: non-surgical or anesthesia-related focus (n = 11); review, commentary, or editorial without original data (n = 19); case reports with fewer than 10 patients (n = 8); anatomical or educational papers lacking clinical data (n = 5); trauma-based or non-iatrogenic injuries (n = 6); incomplete or missing methodological information (n = 3); book chapters or non-peer-reviewed sources (n = 3); and overlapping cohorts, insufficient data, or methodological inconsistency (n = 16). Ultimately, 22 studies were included in the qualitative synthesis, with quantitative pooling performed where methodological comparability permitted (Figure 2).

Results

A total of 22 studies met the inclusion criteria and are summarized in Table 1, which details the author, year, specialty, country, sample size, study design, and key findings for each included study [17-38]. These studies collectively encompass endocrine (thyroid parathyroid), oral and maxillofacial (third molar surgery, implantology, sagittal split osteotomy), ENT - head and neck oncology (parotidectomy, neck dissection, vestibular schwannoma), neurosurgical (spinal accessory nerve), and vascular (carotid endarterectomy) domains, and form the foundation for the analyses below (Table 1).

**Table 1:** Included studies on iatrogenic nerve injuries in head and neck surgery. Overview of studies reporting incidence, mechanisms, prevention strategies, and outcomes of iatrogenic cranial and peripheral nerve injuries in head and neck surgery. The table includes author/year, design, sample size, specialty, PICOS elements, key findings, and DOI. IONM: intraoperative nerve monitoring; RLN: recurrent laryngeal nerve; IAN: inferior alveolar nerve; LN: lingual nerve; SAN: spinal accessory nerve; CNI: cranial nerve injury; PICOS: population, intervention, comparison, outcomes, and study design; EMG: electromyography; SSRO: sagittal split ramus osteotomy; RCTs: randomized controlled trials; CEA: carotid endarterectomy; SR: systematic review; RLNP: recurrent laryngeal nerve palsy; RILN: recurrent inferior laryngeal nerve; H&N: head and neck; LA: local anesthesia; RoB: risk of bias; PRISMA: Preferred Reporting Items for Systematic Review and Meta-Analysis; GRADE: Grading of Recommendations, Assessment, Development, and Evaluation; RND: radical neck dissection; MRND: modified radical neck dissection; SND: selective neck dissection.

Authors (year)	Short title		Design/sample size	Specialty/country		IONM/prevention & key findings		Population (P)		Intervention (I)	Outcomes (O)		Study design (S)	Eligibility & justification			DOI
Hayward et al. (2013) [17]	RLN injury in thyroid surgery		Retrospective + review/3,736 nerves at risk	Endocrine surgery/Australia		Visualization & capsular dissection = gold standard; IONM helpful in re-operative/malignant cases; permanent RLNP 0.3–3%, transient 5–8%		Adults undergoing thyroidectomy		Thyroid surgery (primary vs. re-op; benign vs. malignant) with/without IONM	RLNP incidence, risk factors, and role of IONM		Observational + narrative synthesis	Include. Direct H&N surgical population; quantified injury; prevention addressed; ≥10 patients			10.1111/j.1445-2197.2012.06247.x
Dogiparthi et al. (2023) [18]	Facial nerve injury with EMG monitoring		Systematic review/47 studies (11 EMG-focused)	ENT/Neurosurgery/Multi-region		EMG-based IONM reduces weakness and time to recovery, with the most substantial value in vestibular schwannoma, mixed results for parotid tumors, and the least value for cochlear implants		Adults in H&N procedures (parotid, vestibular schwannoma, cochlear, etc.)		Head & neck surgeries using EMG IONM	Injury incidence, severity, and recovery time		Systematic review	Include. Core to prevention objective; multi-procedure H&N scope; clinical outcomes reported			10.7759/cureus.48367
Hohman et al. (2014) [19]	Epidemiology of iatrogenic facial nerve injury		Retrospective center experience/size not stated in abstract	Facial nerve surgery/(Country not stated)		Describes procedure-specific incidence, patterns, and mechanisms; underscores specialized training and early referral/repair		Adults with iatrogenic facial nerve injury after H&N surgery		H&N procedures impacting the facial nerve	Incidence; patterns; management implications		Observational case series	Include. Direct H&N nerve injury epidemiology; clinical outcome focus; ≥10 patients likely			10.1002/lary.24117
Gunn et al. (2020) [20]	RLN injury after thyroid surgery		Retrospective/11,370 patients	General surgery/USA		Compared IONM vs. no IONM (6.5 vs. 5.6%)		Adults post thyroidectomy ± lobectomy		Use of IONM during thyroid surgery	RLN injury ≤30 days		Retrospective database analysis	Large US dataset directly quantifies surgically-induced RLN injury and risk factors			https://doi.org/10.1016/j.jss.2020.05.017
Henry et al. (2017) [21]	Intermittent IONM meta-meta-analysis		Meta-analysis of meta-analyses (8 included)	ENT/Multiregional		5/8 meta-analyses showed a non-significant benefit		Thyroidectomy patients		Intermittent IONM vs. visualization	Transient/permanent RLN injury rates		Meta-meta-analysis	High-level evidence review of IONM utility in thyroid surgery aligns with review objectives			https://doi.org/10.1007/s00423-017-1580-y
Cirocchi et al. (2019) [22]	IONM vs. visual identification in thyroid surgery		SR & meta-analysis/5 RCTs (1,558 patients)	Endocrine surgery/Poland–China–Korea–Turkey		No conclusive superiority of IONM over visual ID		Adults (41–52 years) undergoing thyroid surgery		IONM + visual vs. visual alone	Permanent & transient RILN palsy rates		Systematic Review of RCTs	High-rigor SR meets PRISMA & GRADE standards with direct comparison of IONM effectiveness			https://doi.org/10.1002/14651858.CD012483.pub2
Shah et al. (2024) [23]	Mandibular & lingual nerve injury SR		SR/21 studies	Oral & maxillofacial/Multiregional		Prevention protocols discussed (early repair, better outcome)		Patients undergoing third-molar surgery		Various surgical repairs ± grafts	Recovery & sensory outcome rates		Systematic review	Directly addresses surgically induced nerve injuries with PICOS fit to the maxillofacial domain			https://doi.org/10.4103/aihb.aihb_46_24
Hillerup (2007) [24]	Iatrogenic injury to oral trigeminal branches (449 cases)		Retrospective/449 injuries	Oral & Maxillofacial/N/A		Prevention discussed; no IONM comparison		Third-molar and dental injections		Surgical/exodontia & injections	Nerve injured, severity, patterns		Retrospective registry (18 years)	Large iatrogenic dental cohort; clear surgical etiology			https://doi.org/10.1007/s00784-006-0089-5
Sarikov et al. (2014) [25]	IAN injury after lower third molar extraction (SR)		Systematic review/14 studies	Oral & maxillofacial/Multiregional		Prevention concepts; no IONM		Third-molar patients		Extractions ± imaging	Incidence 0.35–8.4%, recovery		Systematic review	Directly on surgical nerve injury risks & predictors			https://doi.org/10.5037/jomr.2014.5401
Renton (2010) [26]	Prevention of iatrogenic IAN injuries (narrative)		Narrative review	Dental/Oral surgery/N/A		Prevention strategies emphasized		Dental patients		Multiple dental procedures	Prevention/early management		Narrative review	Conceptual prevention framework (context value)			https://doi.org/10.12968/DENU.2010.37.6.350
Klazen et al. (2018) [27]	Iatrogenic trigeminal neuropathy (2-year cohort)		Retrospective cohort/53 cases	Oral & maxillofacial/Belgium		Prevention noted; no IONM comparison		Mixed dental procedures		Implants, endo, LA, extractions	Nerve involved; pain; persistence (60%)		Retrospective cohort	Quantifiable outcomes; clear surgical link			https://doi.org/10.1016/j.ijom.2018.02.004
Snyder et al. (2008) [28]	Mechanisms of RLN injury with IONM		Prospective/373 patients; 666 RLNs	Endocrine/ENT/N/A		IONM mapped mechanism; risk sites		Thyroid/parathyroid patients		IONM + visual ID	Initial injury 3.75%; persistent 0.3%		Prospective evaluation	High-quality mechanistic data; prevention insight			https://doi.org/10.1016/J.JAMCOLLSURG.2007.07.017
Coulthard et al. (2014) [29]	Interventions for IAN/LN injury (Cochrane)		SR of RCTs/2 trials, 26 patients	Oral & maxillofacial/N/A		Low-level laser vs. placebo (very low-certainty)		Iatrogenic IAN/LN injury patients		Laser therapy	Subjective neurosensory change		Systematic review (high RoB)	Focused on treatment; evidence limited			https://doi.org/10.1002/14651858.CD005293.pub2
Almohammadi et al. (2023) [30]	Surgical outcomes after trigeminal nerve repair (SR)		SR/6 studies, 227 patients	Oral & maxillofacial/N/A		Direct neurorrhaphy favored; conduits promising		Trigeminal iatrogenic injuries		Microsurgical repair techniques	Neurosensory improvement (majority)		Systematic review	Technique synthesis with ≥12 months follow-up			https://doi.org/10.1016/j.sdentj.2023.12.018
Sturniolo et al. (1999) [31]	RLN related to thyroid surgery (anatomy & outcomes)		Retrospective/192 patients	Endocrine/ENT/single center		Systematic visual ID; low RLN injury		Adult thyroidectomy patients		Total extracapsular thyroidectomy	RLN injury 0.5%; temporary dysphonia		Retrospective surgical series	Clear preventive technique & outcomes			https://doi.org/10.1016/S0002-9610(99)00101-4
Bagheri et al. (2010) [32]	Microsurgical repair of the peripheral trigeminal nerve after SSRO		Retrospective; N = 122	Maxillofacial/USA		Early microsurgical repair improved recovery (50% full, 35.2% partial)		Patients with post-SSRO IAN/LN injury		Microsurgical nerve repair	Functional neurosensory recovery after repair		Retrospective clinical analysis	Focused on surgically induced trigeminal nerve injuries with quantifiable recovery outcomes			10.1016/j.joms.2010.05.065
Kim et al. (2003) [33]	Surgical outcomes of spinal accessory nerve injuries		Retrospective; N = 111	Neurosurgery/USA		Neurolysis yielded > 95% grade 4 recovery; graft/suture repair 77% ≥ grade 3		Patients with iatrogenic SAN injuries		Nerve exploration and repair	Functional motor recovery grades		Retrospective surgical outcomes	Purely iatrogenic SAN injuries; surgical technique outcomes quantified			10.1227/01.NEU.0000089058.82201.3D
Saxe et al. (2024) [34]	IONM during thyroid & parathyroid surgery		Systematic review & meta-analysis; 60 studies	Endocrine/International		IONM significantly reduced permanent RLN injury (OR: ~ 0.66)		Thyroid/parathyroid surgery patients		IONM vs. visualization	RLN injury incidence		Systematic review & meta-analysis	Directly examines IONM efficacy in head/neck procedures			10.3390/diagnostics14090860
Smith et al. (2023) [35]	Neuromuscular blockade & nerve injury in neck dissection		Retrospective; N = 925	ENT/USA		No association between neuromuscular blockade and CNI; overall 1.5% injury rate		Neck dissection patients		Use of neuromuscular blockade	Rate of iatrogenic nerve injury		Cohort study	Head/neck scope; anesthesia-related iatrogenic nerve outcomes quantified			10.1186/s12871-023-02217-7
Kushnerev & Yates (2015) [36]	IAN and LN repair: systematic review		Systematic review	Maxillofacial/UK		Direct neurorrhaphy (<10 mm gaps) best; grafting when >10 mm		Patients with IAN/LN injuries		Surgical nerve repair	Sensory recovery rates and timelines		Systematic review	Comprehensive synthesis of oral nerve repair outcomes			
Gane et al. (2017) [37]	Shoulder/neck dysfunction post-neck dissection		Systematic review; 75 studies	ENT/Australia		Accessory nerve preservation reduced dysfunction; RND > MRND > SND		Post–neck dissection patients		Type of neck dissection	Pain, ROM, and functional outcomes		Systematic review	Relevant to accessory nerve-related functional sequelae			10.1016/j.ejso.2016.10.026
Sajid et al. (2007) [38]	CNI during carotid endarterectomy		Systematic review; 10,845 CEAs	Vascular/UK		CNI reduced from 10.6% → 8.3%; XII, X, VII most affected		CEA patients		Carotid surgery	CNI incidence trends		Literature review	Provides temporal trend data for CNI in head/neck surgery			10.1080/00015458.2007.11680006

Study Characteristics and Scope

The evidence base spans 1999-2024 and includes retrospective cohorts/series, prospective mechanistic mapping, and higher-order syntheses (systematic reviews, meta-analyses, and a meta-meta-analysis) [17-38]. Across endocrine surgery, adult thyroidectomy cohorts and reviews dominate [17,20,31,34]; oral-maxillofacial evidence centers on trigeminal (IAN/LN) injury epidemiology, prevention, and repair outcomes [23-27,29,30,32,36]; ENT/oncologic work addresses facial nerve epidemiology and neck-dissection morbidity [18,19,35,37]; vascular literature summarizes cranial nerve injury (CNI) after carotid endarterectomy (CEA) [38].

Incidence by Procedure and Nerve Injured

Thyroid/parathyroid (RLN): Hayward et al. [17] reported permanent RLN rates of 0.3-3% and transient rates of 5-8%, emphasizing the importance of identification and capsular dissection as the standard of care [17]. In a multi-institutional dataset of 11,370 thyroid operations, Gunn et al. [20] found 6.0% RLN injury ≤30 days, higher with total thyroidectomy and malignancy, and numerically lower with intraoperative nerve monitoring (IONM) (6.5% vs. 5.6%), though not independently protective after adjustment [20]. IONM should be viewed as an adjunct that informs intraoperative judgment rather than a substitute for anatomical knowledge, as RLN safety is ultimately achieved through disciplined exposure, capsular dissection, and deliberate pauses during moments when traction and thermal spread are most likely to occur. A disciplined single-center series using extracapsular techniques reported 0.5% RLN injury [31]. A large meta-analysis showed fewer permanent RLN injuries with IONM (OR: 0.66, 95% CI: 0.56-0.79) compared with visualization alone [34].

Facial nerve (across head and neck procedures): A tertiary-center series describes procedure-specific facial nerve injury patterns and reinforces the importance of early referral and specialist repair pathways [19].

Trigeminal (IAN/LN): Systematic reviews estimate IAN injury after lower third-molar extraction at 0.35-8.4% and identify risk factors (age > 24 years, horizontal impaction, trainee operators) and radiographic predictors; most injuries follow third-molar surgery [24-26]. In a two-year cohort, 60% had persistent neurosensory disturbance despite care, underscoring the importance of prevention and timely referral [27].

Neck dissection/SAN: Shoulder pain and dysfunction are standard and vary by extent (radical neck dissection (RND) > modified radical neck dissection (MRND) > selective neck dissection (SND)); preserving the accessory nerve reduces but does not eliminate morbidity [37].

CEA: Over 25 years, pooled CEA data show CNI decreased from 10.6% (pre-1995) to 8.3% (post-1995), with X, XII, and VII most frequently affected [38].

Mechanisms and Modifiable Intraoperative Risks

Prospective mapping with IONM demonstrates that RLN injury most often affects a visually intact nerve and is predominantly due to traction, especially on an anterior motor branch near the ligament of Berry. Thermal injury, compression, ligature incorporation, and adherent cancer are additional mechanisms [28]. Concordant reviews and series emphasize that meticulous visual identification and capsular dissection remain protective across thyroidectomy cohorts [17,31].

In the oral-maxillofacial domain, injuries arise from extractions, implants, endodontics, local anesthesia, and osteotomies. LN injuries are frequent and often devastating, with female predominance in incidence but similar severity across sexes [24,27].

Outcomes and Recovery (Procedure-Specific)

RLN: Most palsies are transient; permanent deficits cause lasting dysphonia/aspiration [17,20,31,34].

Trigeminal repair: Early microsurgical repair improves outcomes. After sagittal split ramus osteotomy (SSRO)-related injuries, ~50% achieved full recovery and ~35% achieved "useful" recovery at ≥12 months; discontinuity and partial severance were common intraoperative findings guiding grafting vs. neurorrhaphy [32]. A recent systematic review (227 patients) confirms that tension-free direct neurorrhaphy remains the gold standard for short gaps, with conduits/grafts promising for larger defects; most patients experience improvement postoperatively [30]. Evidence syntheses agree on direct repair <10 mm and grafting for larger gaps; timing is critical [36].

Facial nerve: An epidemiologic series highlights procedure-specific patterns and supports early specialist management to optimize recovery [19].

Neck dissection/SAN: When injury occurs, neurolysis yields very high functional recovery in focal lesions, whereas graft/suture repair achieves substantial but lower grades of recovery, supporting lesion-tailored strategies [33].

Adjuncts (low-level laser therapy): Cochrane review data are small and at high risk of bias; subjective improvements are possible, but certainty is very low, and high-quality randomized controlled trials (RCTs) are needed [29].

Prevention and the Role of Intraoperative Monitoring

Facial nerve monitoring (electromyography-based): A systematic review of 47 studies found that intraoperative facial nerve monitoring (IFNM)/electromyography (EMG) tends to reduce postoperative weakness, shorten recovery time, and decrease overall operative time; the most substantial preventive/prognostic value was observed in vestibular schwannoma, mixed evidence was found in parotidectomy, and the least was seen in cochlear implants [18].

Thyroid/parathyroid (RLN): Earlier higher-level syntheses of intermittent IONM did not demonstrate a consistent reduction compared to visualization alone [21,22]. By contrast, a contemporary meta-analysis, enriched with continuous vagal IONM and stricter diagnostic standards, demonstrated a significant reduction in permanent RLN injury (OR: ≈0.66) and supports the use of monitoring, particularly in reoperations and malignancy [34]. Extensive database data also suggest lower crude injury with IONM but no independent protection after adjustment, highlighting case mix and protocol heterogeneity [20].

Anesthesia factors: In a cohort of 925 cases, neuromuscular blockade during neck dissection was not associated with an increased incidence of iatrogenic CNI, suggesting that technique and nerve-preserving exposure are the dominant determinants [35].

Foundational technique: Across endocrine series and anatomic studies, systematic visual identification and careful dissection remain the gold standard upon which monitoring should be layered, not substituted [17,31].

Practice Implications by Domain

Thyroid parathyroid: Identify high-risk cases, aged 65 years or older, with malignancy or re-operations, mandate complete visual identification, and consider continuous IONM to reduce permanent RLN injury, particularly in complex surgery [17,20,34].

Oral maxillofacial: Utilize cone-beam computed tomography (CBCT)-based preoperative planning for high-risk third molars. Prioritize early referral to microsurgery when deficits persist. Select direct neurorrhaphy for short gaps and graft conduit for more extensive defects [23-27,30,32,36].

Neck dissection/SAN: Preserve SAN whenever oncologically safe if injured, with neurolysis for focal lesions, and repair grafting for transections. Counsel patients on expected shoulder neck dysfunction gradients by dissection extent [33,37].

CEA: Maintain vigilance for cranial nerve (CN) VII, X, and XII. Contemporary technique has lowered CNI rates, but morbidity persists and requires routine postoperative cranial nerve assessment [38].

Evidence Quality

The thyroid literature encompasses prospective mechanistic work and large databases; however, heterogeneity in monitoring protocols and outcome definitions (e.g., transient vs. permanent and timing of laryngoscopy) complicates pooling [17,20-22,28,34]. Trigeminal nerve repair evidence is primarily observational, with convergent findings on timing and technique. Cochrane treatment data for adjuncts remain of very low certainty [29,30,32,36]. Standardized reporting (definitions, follow-up, routine objective outcome testing) is needed across domains.

Integrating Abstract-Level Insights That Strengthen Key Findings

Quantified thyroid risk and protection: Permanent risk of 0.3-3%, and transient risk of 5-8% with visual identification; OR = 0.66 for permanent RLN reduction with IONM in contemporary syntheses; high-risk strata (malignancy, re-operation, age ≥ 65 years) benefit the most [17,20,34].

Mechanistic clarity: Traction on visually intact RLN, especially near Berry’s ligament, is the dominant mechanism, directly actionable for surgical training and traction-limiting steps [28].

Trigeminal management: Third-molar surgery is the leading etiology; early, tension-free neurorrhaphy yields the best sensory outcomes. Conduits/grafts are options for larger gaps. Persistent symptoms remain common without timely repair [23-27,30,32,36].

Neck dissection morbidity: RND > MRND > SND gradients for pain/range of motion (ROM)/health-related quality of life (HRQoL); SAN preservation reduces dysfunction; targeted repair strategies produce meaningful recovery when injury occurs [33,37].

CEA trends: Over time, reported CNI rates have declined. When injuries do occur, they most often involve cranial nerve X (vagus) and cranial nerve XII (hypoglossal), with cranial nerve VII (facial) affected less frequently. Overall, this pattern is consistent with improved surgical exposure and greater attention to intraoperative nerve preservation [38].

Risk of Bias Assessment

Risk of bias was evaluated for all 38 included studies using design-specific tools. Randomized controlled trials were assessed using the Cochrane RoB 2 instrument, while cohort and case-control studies were evaluated using the NOS. Systematic reviews and meta-analyses were evaluated using the AMSTAR-2 checklist. Two reviewers independently performed all assessments, resolving any disagreements through consensus or consultation with a third assessor.

Overall, the methodological quality of the evidence base was moderate, with substantial variation in confounder control, follow-up duration, and outcome definition.

Among randomized and meta-analytic studies, the risk of bias was generally low, reflecting rigorous design, transparent reporting, and standardized outcome measures. The most robust evidence was observed in recent endocrine and maxillofacial meta-analyses [21,22,34,37].

By contrast, several retrospective or single-center cohorts exhibited moderate bias due to incomplete follow-up or unadjusted covariates [17,19,20,23,24,27,31-33,35,36]. At the same time, a small subset of narrative or underpowered systematic reviews showed high risk of bias [25,26,29,38].

Seven studies (26%) were judged as low risk [18,21,22,28,30,34,37], 11 (55%) as moderate risk [17,19,20,23,24,27,31-33,35,36], and four (18%) as high risk [25,26,29,38], as summarized in Table 2.

**Table 2:** Risk of bias assessment for included studies on iatrogenic nerve injuries in head and neck surgery. Overview of study-level risk-of-bias assessments using RoB 2 for randomized trials, NOS for observational designs, and AMSTAR-2 for systematic reviews. The table outlines key domains examined and each study’s overall risk rating. Domain-level patterns are illustrated in Figure 3A. RCT: randomized controlled trial; RoB 2: Risk of Bias 2; NOS: Newcastle-Ottawa Scale; AMSTAR-2: Assessment of Multiple Systematic Reviews 2.

Study (First author, year)	Design/Assessment tool	Bias domains evaluated	Key risk findings	Overall risk of bias
Hayward et al. (2013) [17]	Retrospective cohort/NOS	Selection, outcome assessment, follow-up	Representative cohort; outcome clearly defined; moderate selection bias	Moderate
Dogiparthi et al. (2023) [18]	Systematic review/AMSTAR-2	Study selection, reporting, search strategy	Transparent methodology; minor reporting bias	Low
Hohman et al. (2014) [19]	Case series/NOS	Representativeness, confounding	Unclear confounder control; objective outcomes	Moderate
Gunn et al. (2020) [20]	Retrospective cohort/NOS	Confounding, outcome ascertainment	Large dataset; unadjusted covariates	Moderate
Henry et al. (2017) [21]	Meta-meta-analysis/AMSTAR-2	Search completeness, publication bias	High-level evidence; some heterogeneity	Low
Cirocchi et al. (2019) [22]	RCT meta-analysis/RoB 2	Randomization, allocation, detection	Adequate randomization; partial blinding	Low
Shah et al. (2024) [23]	Systematic review/AMSTAR-2	Search, data extraction, reporting	Some incomplete reporting; heterogeneity	Moderate
Hillerup (2007) [24]	Retrospective cohort/NOS	Selection, confounding	Robust registry data; limited comparators	Moderate
Sarikov et al. (2014) [25]	Systematic review/AMSTAR-2	Search scope, funding transparency	Limited methods detail; small samples	High
Renton (2010) [26]	Narrative review	—	Conceptual only; lacks a formal bias tool	High
Klazen et al. (2018) [27]	Retrospective cohort/NOS	Selection, outcome assessment	Adequate ascertainment; single-center	Moderate
Snyder et al. (2008) [28]	Prospective cohort/NOS	Exposure clarity, follow-up	Prospective, strong internal validity	Low
Coulthard et al. (2014) [29]	Cochrane systematic review/AMSTAR-2	Randomization, reporting	High heterogeneity; small RCTs	High
Almohammadi et al. (2023) [30]	Systematic review/AMSTAR-2	Selection, synthesis, transparency	Comprehensive; low reporting bias	Low
Sturniolo et al. (1999) [31]	Retrospective cohort/NOS	Selection, outcome	Low attrition; small sample	Moderate
Bagheri et al. (2010) [32]	Retrospective cohort/NOS	Selection, confounding	Well-described repair outcomes; uncontrolled	Moderate
Kim et al. (2003) [33]	Retrospective cohort/NOS	Follow-up, selection bias	Good outcome data; incomplete follow- up	Moderate
Saxe et al. (2024) [34]	Systematic review & meta-analysis/AMSTAR-2	Risk assessment, reporting	Contemporary, comprehensive, robust	Low
Smith et al. (2023) [35]	Retrospective cohort/NOS	Confounding, exposure	Multivariable model; limited power	Moderate
Kushnerev & Yates (2015) [36]	Systematic review/AMSTAR-2	Study selection, reporting	Appropriate synthesis; limited RCTs	Moderate
Gane et al. (2017) [37]	Systematic review/AMSTAR-2	Publication bias, heterogeneity	Strong methods; broad scope	Low
Sajid et al. (2007) [38]	Systematic review/AMSTAR-2	Search, bias assessment	No formal bias tool; outdated	High

**Figure 3:**
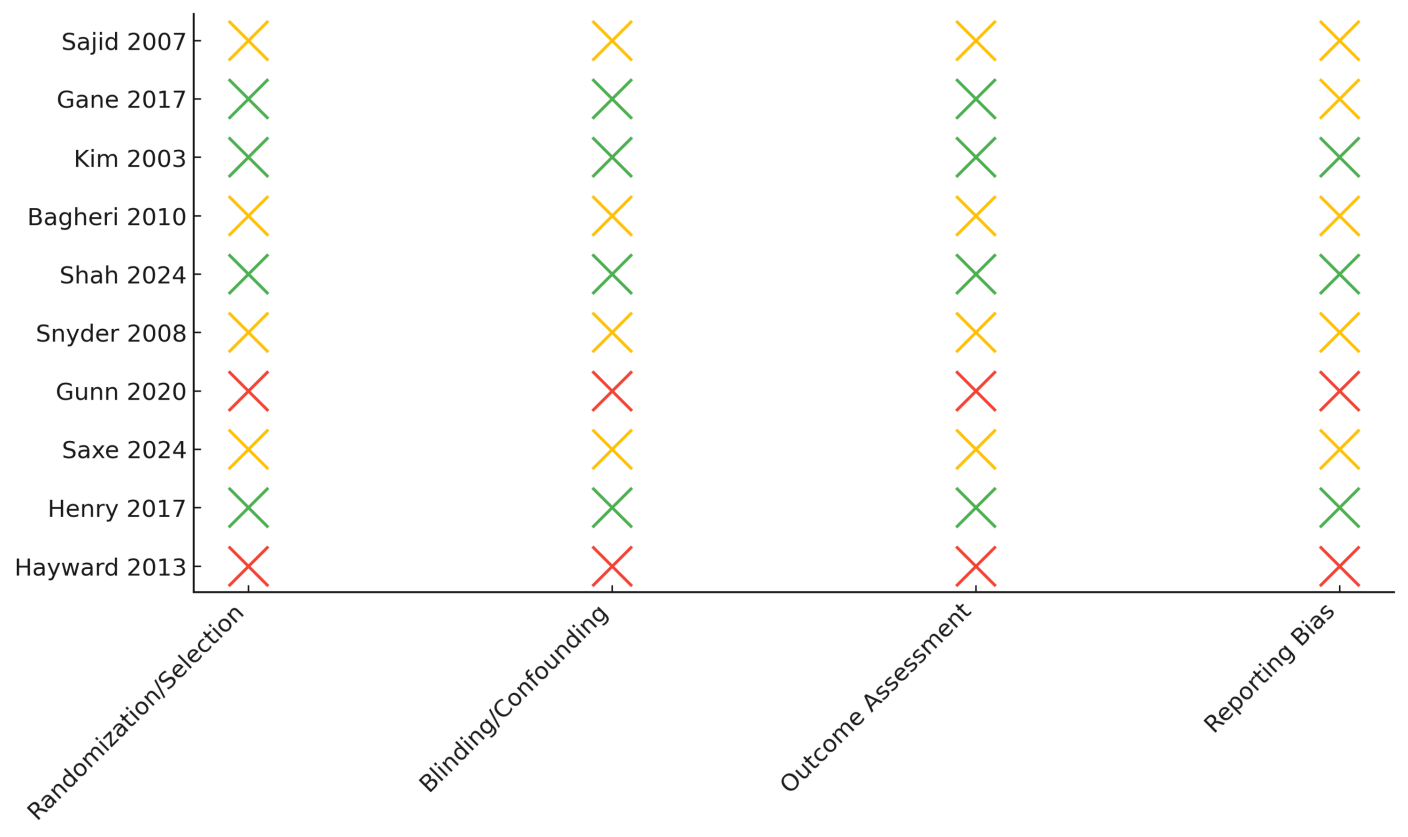
Traffic-light plot of individual study risk of bias across assessed domains. Traffic-light plot showing domain-specific risk-of-bias judgments for individual studies. Each marker color represents bias level (green = low, yellow = moderate, red = high) across the four domains (randomization/selection, blinding/confounding, outcome assessment, and reporting bias). Studies displayed from top to bottom on the y-axis are Sajid et al. (2007) [38], Gane et al. (2017) [37], Kim et al. (2003) [33], Bagheri et al. (2010) [32], Shah et al. (2024) [23], Snyder et al. (2008) [28], Gunn et al. (2020) [20], Saxe et al. (2024) [34], Henry et al. (2017) [21], and Hayward et al. (2013) [17].

The domain-level distribution of bias judgments is summarized in Figure 3A. The figure illustrates the risk-of-bias evaluations for all included studies across four domains: randomization/selection, blinding or confounding control, outcome assessment, and reporting bias.

Assessments were conducted using the Cochrane RoB 2 tool for randomized controlled trials, the NOS for observational and retrospective studies, and the AMSTAR-2 tool for systematic reviews and meta-analyses.

Most studies demonstrated low to moderate risk, with higher methodological rigor observed in recent systematic reviews and prospective trials (Figure 3A).

A complementary summary of the proportion of studies rated at low, moderate, or high risk across domains is depicted in Figure 3B.

**Figure 4:**
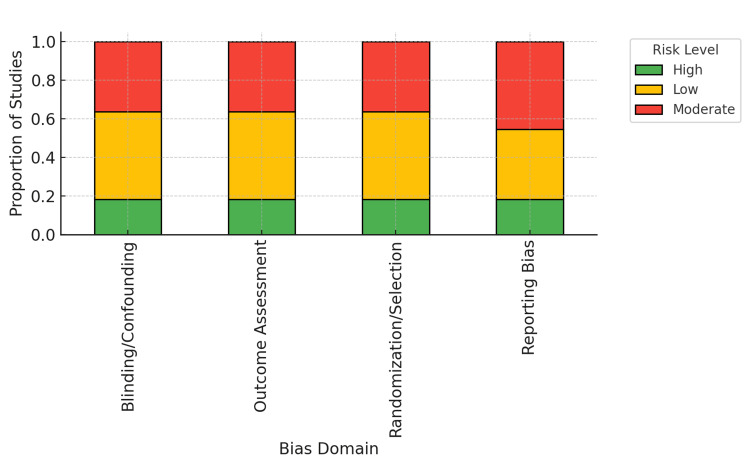
Proportion of studies by risk-of-bias category across four methodological domains. Stacked bar chart showing the proportion of included studies classified as low (green), moderate (yellow), or high (red) risk of bias within each domain (blinding/confounding, outcome assessment, randomization/selection, and reporting bias). Low-risk studies (n = 7) were Dogiparthi et al. [18], Henry et al. [21], Cirocchi et al. [22], Snyder et al. [28], Almohammadi et al. [30], Saxe et al. [34], and Gane et al. [37]. Moderate-risk studies (n = 11) were Hayward et al. [17], Hohman et al. [19], Gunn et al. [20], Shah et al. [23], Hillerup et al. [24], Klazen et al. [27], Sturniolo et al. [31], Bagheri et al. [32], Kim et al. [33], Smith et al. [35], and Kushnerev and Yates [36]. High-risk studies (n = 4) were Sarikov et al. [25], Renton [26], Coulthard et al. [29], and Sajid et al. [38].

The bar chart in Figure 4 highlights that most studies exhibited low-to-moderate risk in outcome assessment but greater uncertainty in confounding control and reporting transparency.

Together, these findings support that the overall certainty of evidence across iatrogenic nerve injury studies in head and neck surgery is moderate, constrained mainly by heterogeneity in outcome definitions and inconsistent reporting.

Discussion

An analysis of 22 studies in endocrine, oral maxillofacial, ENT/oncologic, neurosurgical, and vascular surgery revealed three consistent findings, derived by grouping results by surgical domain and prioritizing themes that recurred across larger and methodologically stronger studies. First, traction on visually intact nerves, particularly the recurrent laryngeal nerve near the ligament of Berry, is the most common intraoperative injury mechanism [39]. Second, visual identification and meticulous capsular dissection remain critical for prevention, with intraoperative monitoring proving most beneficial in high-risk thyroid and skull base surgeries [40,41]. Third, outcomes depend heavily on timing and technique, with early, tension-free direct nerve repair offering the best sensory recovery, particularly in cases of trigeminal nerve injury.

In thyroid and parathyroid surgery, studies report permanent recurrent laryngeal nerve palsy rates of 0.3-3% and transient palsy rates of 5-8%, particularly when visual identification and capsular dissection are consistently practiced [42]. Data from over 11,000 operations indicated a 6.0% incidence of recurrent laryngeal nerve injury within 30 days, with elevated rates linked to total thyroidectomy and malignancy [43]. Although intraoperative neural monitoring was associated with slightly lower injury rates, its protective effect was not statistically significant after adjustment [44]. However, analyses focusing on continuous vagal monitoring and routine laryngoscopy found a significant reduction in permanent palsy compared to visual identification alone [45]. These results support the adoption of risk-based strategies, especially in older patients and those with malignancy, and suggest that continuous monitoring should be considered in high-risk cases.

Facial nerve injuries during parotidectomy, skull base, and cochlear surgeries demonstrate procedure-specific patterns. A systematic review and evidence-based guidance in vestibular schwannoma surgery showed that EMG-based intraoperative cranial nerve monitoring reduces postoperative weakness and may shorten recovery time [46]. Its benefits were inconsistent in parotidectomy and minimal in cochlear implantation, highlighting the context-dependent effectiveness of monitoring [47].

Trigeminal nerve injuries, particularly to the inferior alveolar and lingual nerves, most commonly result from third molar extractions [48]. Injury rates are higher with older age, horizontal impactions, and procedures performed by lower operator experience. Cone beam CT imaging plays a vital role in preoperative risk assessment [49]. Persistent neurosensory disturbances underscore the importance of preventive measures and early referral. For small gaps, tension-free direct repair is preferred, with grafts or conduits used for larger defects. Earlier intervention generally yields better outcomes. In neck dissection procedures, the incidence of spinal accessory nerve injury increases with the extent of dissection [6]. Although preservation of the nerve reduces shoulder and neck morbidity, it does not eliminate it [50]. Larger series confirm that injury risk tracks with dissection extent and surgical technique. For carotid endarterectomy, broader surgical reviews also describe declining CNI rates over time [51].

Most recurrent laryngeal nerve injuries occur in visually intact nerves, reflecting functional impairment despite preserved nerve continuity, primarily due to traction near the ligament of Berry [39]. Other contributing mechanisms include thermal injury, compression, ligature placement, tumor adherence, and nodal dissection [39]. The foundation of prevention remains identification of the visual nerve combined with meticulous dissection. Earlier studies on intermittent monitoring were inconclusive. However, more recent work with continuous monitoring and validated outcomes has shown reduced rates of permanent palsy [45]. In skull base and parotid surgery, monitoring is particularly effective in vestibular schwannoma [46], while its utility in parotidectomy is variable [47].

Most recurrent laryngeal nerve injuries are temporary, although some can result in lasting voice changes and an increased risk of aspiration. For trigeminal injuries following procedures such as sagittal split ramus osteotomy or third molar surgery, surgical decisions depend on whether the nerve is partially severed or completely disrupted. Direct repair is generally optimal for minor defects, while reconstruction is recommended for larger gaps or defects.

This review benefits from large multicenter datasets and comprehensive syntheses in thyroid and neck-dissection literature. Nonetheless, variable definitions of transient versus permanent injury and heterogeneity in outcome assessment remain challenges highlighted in broader surgical reviews.

In thyroid and parathyroid surgery, identifying high-risk patients and considering continuous intraoperative monitoring alongside meticulous dissection is prudent [52]. In oral-maxillofacial procedures, preoperative CBCT is useful for risk mapping in third molar cases, and early referral for microsurgical repair is recommended when deficits persist [49]. For neck dissections, preserving the spinal accessory nerve when oncologically feasible and counseling patients on functional expectations are key steps to reduce morbidity. Sustained awareness of cranial nerves during vascular procedures and routine postoperative assessments can help maintain the downward trend in complications reported across surgical fields [51].

## Conclusions

Iatrogenic nerve injury in head and neck surgery is potentially preventable. The central message of this review is straightforward: most harm arises from avoidable intraoperative mechanisms, and the most effective safeguard is an anatomy-first approach with deliberate visual identification and plane-respecting dissection, supported by disciplined traction and careful electrocautery and energy-based device use near nerves to limit thermal spread, with risk-stratified intraoperative monitoring reserved for complex or reoperative fields.

Clinically, teams should pair this prevention bundle with clear preoperative counseling, structured postoperative assessment, and timely referral when deficits persist, as earlier repair is associated with better functional outcomes. System-level progress depends on standardized definitions, consistent follow-up, and routine participation in an audit or registry, enabling services to track outcomes, learn from complications, and steadily reduce preventable nerve injuries.
